# Practical training in medical sociology “Analysis of social environments of living quarters”. A field experiment during the COVID-19 pandemic

**DOI:** 10.3205/zma001423

**Published:** 2021-01-28

**Authors:** Enno Swart, Annemarie Feißel, Aiad Hasoon, Madlen Hörold, Heike Hupach, Uwe Matterne, Katharina Piontek, Matthes Schaefer, Christoph Stallmann, Marco Strecker, Christian Apfelbacher

**Affiliations:** 1 Otto-von-Guericke-Universität Magdeburg, Medizinische Fakultät, Institut für Sozialmedizin und Gesundheitssystemforschung, Magdeburg, Germany

**Keywords:** medical sociology, social epidemiology, neighborhood, social environment analysis

## Abstract

**Background:** In the summer semester 2020, a new format was introduced at the Otto-von-Guericke-University Magdeburg for first-year medical students in the subject of medical sociology with a neighborhood-related social environment analysis.

**Didactic approach:** Due to the COVID-19 pandemic, the didactic concept had to be changed at short notice from seminar group-related fieldwork in different districts of Magdeburg to individual work at the place of study or home, supplemented by accompanying online offers. The students were asked to characterize their neighborhood in terms of quality of life, health and illness as well as medical care by means of interviews with inhabitants of their immediate living environment, a neighborhood inspection with the taking of photographs and an analysis of official secondary data. The aim was to gain initial experience in scientific work (data collection, presentation and interpretation of results, as well as reporting). An evaluation of this new course and conclusions derived from it for its further development will be reported.

**Evaluation:** 51 percent of the students participated in an evaluation of the course. The clear majority rated the internship as “good” or “very good”. As a suggestion for improvement, the desire for optional supplementary individual counseling and better formal preparation for the performance assessments were expressed several times. Two thirds of those surveyed consider the online teaching format to be useful even in post-pandemic times.

## Zusammenfassung

**Hintergrund: **Im Sommersemester 2020 wurde an der Otto-von-Guericke-Universität Magdeburg für Studierende der Humanmedizin im ersten Studienjahr im Fach Medizinische Soziologie mit einer quartierbezogenen Sozialraumanalyse ein neues Format eingeführt.

**Didaktischer Ansatz: **Aufgrund der COVID-19-Pandemie musste das didaktische Konzept kurzfristig von einer seminargruppenbezogenen Feldarbeit in verschiedenen Magdeburger Stadtteilen auf eine individuelle Arbeit am Studien- oder Heimatort umgestellt werden, ergänzt um begleitende online-Angebote. Die Studierenden sollten mittels Interviews mit Einwohner*innen ihres unmittelbaren Wohnumfelds, einer Quartierbegehung mit Anfertigung von Fotoaufnahmen sowie einer Analyse von amtlichen Sekundärdaten eine Charakterisierung ihres Quartiers mit Blick auf Lebensqualität, Gesundheit und Krankheit sowie medizinische Versorgung vornehmen. Dabei sollten erste eigene Erfahrungen im wissenschaftlichen Arbeiten gesammelt werden (Datengewinnung, Ergebnisdarstellung und Interpretation sowie Berichterstattung). Es wird über die Evaluation dieses neuen Lehrangebots und daraus abgeleiteten Schlussfolgerungen zu dessen Weiterentwicklung berichtet.

**Evaluation: **51 Prozent der Studierenden nahmen an einer Evaluation der Lehrveranstaltung teil. Die deutliche Mehrheit bewertet das Praktikum mit „gut“ oder „sehr gut“. Als Verbesserungsvorschlag wird mehrfach der Wunsch nach einer optional ergänzenden individuellen Beratung und einer besseren formalen Vorbereitung auf die Leistungsnachweise geäußert. Zwei Drittel der Befragten erachten das online-Lehrformat auch in Nach-Pandemie-Zeiten für sinnvoll.

## Background

Theories, models and empirical evidence on the relationship between the social status of individuals and their health [1], [2], [3], [4] are an integral part of the preclinical teaching of medical sociology at the Medical Faculty of the Otto-von-Guericke-University Magdeburg (OvGU), under the responsibility of the Institute of Social Medicine and Health System Research (ISMG). The course in medical sociology focuses on the teaching of qualitative and quantitative methods of social science and epidemiology. The following short report describes the connection of both topics in a practical course of medical sociology under the concrete conditions of the COVID-19 pandemic.

## Didactic approach

The influence of sociodemographic and socioeconomic factors on health, health behavior, and the development and course of disease is the subject of courses in medical sociology (first year), supplemented by discussions of concepts and projects in health promotion and prevention [5], [6]. The setting approach [7] has not yet been broken down to the level of the immediate living environment (“neighborhood”) in which everyday activities take place (living, shopping, doctor's visits, leisure time, etc.) and the question of whether and, if so, how the neighborhood act as an independent determinant of the quality of life, satisfaction and health of the residents [8], [9], [10]. In the summer semester 2020, this topic was included as an object of a practical course in medical sociology, combined with previously theoretically taught methodological techniques.

The original teaching concept provided for a field phase for ten seminar groups in different parts of Magdeburg. This phase was to include an inspection, a questionnaire survey and qualitative interviews with residents and with employees of the health and social services (doctors, pharmacists, etc.) as well as a presentation of results in the form of a report and a scientific poster. In this internship, in addition to known models of the influence of social status on the health of individuals, the residential area should be considered as a possible further distinct determinant of the health of its inhabitants. Furthermore, basic techniques of scientific work (research, study planning, analysis, reporting) should be practiced for the students’ first time during the course of the study.

In view of the COVID-19 pandemic, this concept had to be changed at short notice. The students were now required to write an individual study thesis and to complete three subtasks at their place of study in Magdeburg or at their home town:

1. a description of the neighborhood on the basis of accessible secondary data [11],
2. an individual inspection of the neighborhood with the production of photo and film material [12],
3. an execution of at least one interview (personal/telephone) in their immediate personal environment (e.g. household roommate, neighbor).

The students should write down their experiences in a summarizing report (approx. 8,000 to 10,000 characters) and an abstract, including an individual summary of their newly gained insights in terms of content and methodology. Prior to the field phase, an introductory plenary session and two online meetings (zoom conferences) with the seminar groups’ lecturer were held online.

The original learning objectives were further tailored to the setting of a purely online event:

- Consolidation of basic concepts of social epidemiology
- Research, preparation and interpretation of secondary data (here: data of official statistics)
- Practice and evaluation of qualitative research methods
- Recognizing the association between social situation, living environment and health
- Gaining first experiences in planning and implementation of scientific work
- Practice of scientific reporting (abstract, report)

## Experience in implementation of the course

The internship could be carried out within the intended framework after a short-term change of the didactic concept. The majority of the students (60%) carried out the social environment analysis at their home town. The individual reports largely corresponded to the content and formal requirements. The students received summary generic feedback on their reports, which addressed frequently occurring deficiencies in content, methodology, and form (e.g., lack of internal structure, too brief description of the methods, inadequate citation and referencing), A individual feedback was offered but was only used sporadically.

## Evaluation

96 of 188 students (51%) participated in the online evaluation of the internship. The internship was predominantly evaluated positively by the participants; more than half of them gave the grade “1” or “2”. The majority felt sufficiently prepared for the individual field phase through the introductory online events and the materials provided. Nevertheless, about half of the participants would have found helpful a further online introduction to the concrete tasks and the requirements for the written report. The photo and video tour of the neighborhood and the contacting of discussion partners were mostly considered unproblematic. Obtaining official data to describe the socioeconomic structure proved to be difficult for smaller communities. The accessibility of the lecturers was predominantly evaluated as good. Two-thirds of the students participating in the evaluation preferred to carry out this internship as an online event rather than a classroom-based one. Improvements suggested include more targeted preparation of the field phase and more concrete advice on the formal design of the report.

## Conclusion

In view of the COVID-19 pandemic, the short-term change in the format of the neighborhood-related social environment analysis in a Magdeburg district towards a free choice of “neighborhood” has proven itself from the students’ perspective. The possibility of individual time arrangements, the practice of scientific methods and the view of the “importance of the living environment in relation to health” (quote from the evaluation) were positively rated. The numerous comments made by the students help in the further development of the format. Online tools (e.g. confluence, microsoft teams) are available to help students work on tasks in student groups and intensify communication with the lecturers. In the opinion of the students, the new format is also suitable for implementation under COVID-19 teaching conditions that have to be observed in the long term. The evaluation of the course offerings will be continued.

Regardless of the specific format, this internship, together with other courses in medical sociology, is suitable for demonstrating to students the influence of the living environment on health [8], [13], [14], [15], [16] and at the same time offers the opportunity to learn and practice basic scientific working techniques that have been upgraded in the German National Competence-Based Learning Objectives Catalogue for Medicine (NKLM). A consolidation of the learned scientific working techniques is possible in electives of the ISMG in the pre-clinical and clinical sections.

## Competing interests

The authors declare that they have no competing interests. 
